# COVID-19-Related Financial Hardship, Job Loss, and Mental Health Symptoms: Findings from a Cross-Sectional Study in a Rural Agrarian Community in India

**DOI:** 10.3390/ijerph18168647

**Published:** 2021-08-16

**Authors:** Sangeeta Chatterji, Lotus McDougal, Nicole Johns, Mohan Ghule, Namratha Rao, Anita Raj

**Affiliations:** Center on Gender Equity and Health, University of California San Diego, La Jolla, CA 92093, USA; lmcdouga@health.ucsd.edu (L.M.); nejohns@health.ucsd.edu (N.J.); drmohang@gmail.com (M.G.); geh.india@health.ucsd.edu (N.R.); anitaraj@health.ucsd.edu (A.R.)

**Keywords:** COVID-19, mental health, financial hardship, job loss, wage loss, unemployment, depression, anxiety, panic disorder

## Abstract

Several countries, including India, imposed mandatory social distancing, quarantine, and lockdowns to stop the spread of the SARS-CoV-2 virus. Although these measures were effective in curbing the spread of the virus, prolonged social distancing, quarantine, and the resultant economic disruption led to an increase in financial stress and mental health concerns. Prior studies established a link between the first lockdown and an increase in mental health issues. However, few studies investigated the association between post-lockdown financial hardship, job loss, and mental health. In this study, we examined the association between COVID-19-related financial hardship, job loss, and mental health symptoms approximately nine months after the end of the first nationwide lockdown in India. Job loss was associated with higher reporting of mental health symptoms among men (aIRR = 1.16) while financial hardship was associated with poor mental health symptoms among women (aIRR = 1.29). Conversely, social support and government aid were associated with better mental health symptoms among women. Our findings highlight the need for financial assistance and job creation programs to aid families in the recovery process. There is also an urgent need for improving the availability and affordability of mental health services in rural areas.

## 1. Introduction

The COVID-19 pandemic impacted economic activity across the world [1]. India has been severely impacted by the pandemic, with an overburdened health system, federal mismanagement, lack of transparent data, low vaccination rates, and shortages of necessities, such as oxygen and hospital beds [2,3]. Several countries, including India, imposed mandatory social distancing, quarantine, and lockdowns to stop the spread of the virus. While these measures were effective in curbing the spread of the virus, prolonged social distancing, quarantine, and the resultant economic disruption led to an increase in financial stress and mental health concerns [4,5]. A systematic review found high rates of symptoms of anxiety (6–51%), depression (15–48%), post-traumatic stress disorder (7–54%), psychological hardship (34–38%), and stress (8–82%) reported in the general population during the COVID-19 pandemic in China, Spain, Italy, Iran, the United States, Turkey, Nepal, and Denmark [6]. At particular risk of higher levels of adverse mental health were those who were unemployed, younger (≤40 years), female, and students, as well as individuals with a prior history of chronic/psychiatric illness or exposure to COVID-19-related news [6]. A separate systematic review of studies conducted in South Asia reported a pooled prevalence of 41% for anxiety and 34% for depression. Out of 19 studies conducted in India, only 8 surveyed the general population. In these studies, the rates for anxiety ranged from 15 to 42% and depression ranged from 10 to 75% [7]. Two studies with an internet-recruited and -administered survey in India found that individuals reported an increase in anxiety, depression, and stress symptoms during the lockdown. Women, Muslims, business/self-employed, and individuals with poor health status had higher rates of anxiety [8,9]. Concurrently, there was a 68% increase in online news reports of suicidal behavior during the first nationwide lockdown [10]. Individuals who attempted to or committed suicide were more likely to be middle-aged, male, married, and employed.

In addition to these high levels of adverse mental health, the COVID-19 pandemic affected economic security through increased job loss. In India, unemployment rose to a high of 24% in April 2020, the highest rate in the past 30 years, and remained between 6.5 and 9% since the end of the first nationwide lockdown, which took place from March–May 2020 [11]. The gross domestic product (GDP) declined by 7% in 2020, with a 24% decrease in the first quarter of 2020 [12]. A majority of the Indian population is employed in the informal sector, which was severely impacted by the pandemic. The 2017–2018 National Sample Survey (NSSO) reported that 52% of individuals were self-employed, largely in the informal sector, while 25% were employed in casual daily wage work and 23% were salaried [13]. As per the 2011–2012 NSSO, the national average daily wage rate for informal sector workers was INR 277, less than half of the daily wage rate of INR 716 in the formal sector [14]. In addition to this dependency on cheap informal labor, India also lacks social security programs that could buffer individuals from the adverse effects of financial hardship [15]. In a multi-state study, two-thirds of participants, largely employed in the informal sector, lost employment during the lockdown. Women, Muslims, the lower educated, and migrant populations had higher unemployment rates [15]. Another study that surveyed self-identified poverty-stricken families found that 52% of the sample across 15 states lost their jobs and an additional 20% lost wages [16]. State-specific studies noted a job loss rate of 75% in Bihar [17] and 31% in rural Odisha [18].

For many, this job loss was compounded by wage loss. In India, across employment categories, workers’ earnings decreased by 40–50% during the lockdown, with a 55% reduction in earnings among women in rural areas [15]. The agricultural sector was also impacted. While 60% of agricultural workers in rural areas had crops that were ready for harvest and sale, 85% could not harvest or sell the produce or sold it at a reduced price due to a lack of transportation, buyers, machines, and labor [15]. Eighty-five percent of respondents could not pay next month’s rent, 80% reduced their food intake, 47% could not purchase a week’s worth of essentials, and 36% took a loan during the lockdown [15]. Another study found a 65% decline in monthly income, with the top household wealth quintile reporting a 51% reduction as compared to a 71% reduction in the bottom quintile. Forty percent of all families had incurred debt during the lockdown period [16]. In rural Odisha, 93% experienced financial challenges [18].

Prior research established a link between unemployment, financial hardship, and mental health. Three separate systematic reviews, including longitudinal and cohort-based studies, found that individuals who lost their job during a recession and/or experienced financial crises were more likely to report poor health, increased stress, depression, mental hardship, anxiety, and suicidal behaviors [19,20,21]. Evidence also suggests that a recession disproportionately impacts minoritized populations. In two reviews of studies conducted in the United States and Europe, lower-educated, low-income, Black, Latinx, and female populations were more likely to report poor mental health [20,21]. Social security programs can buffer populations from the adverse health impacts of financial crises. A review of studies that were conducted after the 2008 recession found that unemployed individuals who resided in countries with generous unemployment benefits reported better physical and mental health due to increased financial security [22].

Prior studies established a link between the first lockdown and an increase in mental health issues in India. However, few studies investigated the association between pandemic-related job loss and mental health symptoms. Second, most studies focused on the impact of the first lockdown that took place in March–May 2020 and there is a lack of information on the post-lockdown impact of the pandemic on mental health symptoms, job loss, and financial hardship. In this study, we addressed these gaps in the literature by examining the association between COVID-19-related financial hardship, job loss, and mental health symptoms in a rural area in Maharashtra approximately nine months after the end of the first nationwide lockdown in India. Based on prior research, we hypothesized that financial hardship and job loss would be associated with a higher likelihood of reporting mental health symptoms for both women and men. We also hypothesized that social support and government aid (both financial and in kind) would be associated with better mental health for both women and men.

## 2. Materials and Methods

### 2.1. Data and Sample

This study was conducted in Pune District, Maharashtra, which has a rural population of 3.7 million residents across 2000 villages. In rural Pune, the female illiteracy rate is 27%, and the child sex ratio is 833 girls per 1000 boys. Within Pune District, we focused on rural Junnar Taluka (geographic sub-district), which is comprised of 183 villages with a population of 399,000. Maharashtra has the second-highest statewide GDP in India and is an industrial hub. Mumbai, the capital of Maharashtra, is a financial center, while Pune, the second-largest city, is an educational center [23].

We use data from a cross-sectional survey of couples that were previously recruited in 2018–2019 as part of the randomized evaluation of CHARM2 (Counseling Husbands and Wives to Achieve Reproductive Health and Marital Equity 2), which was a multi-component, gender-synchronized, family planning intervention [24]. CHARM2 was designed to improve contraceptive choice, reduce unintended pregnancy, and reduce marital sexual violence. Participants included male–female couples that were enrolled when the women were 18–29 years, neither partner was sterilized, had resided in the village together for 3 months, were planning to stay in the current place of residence for at least 2 years, were fluent in Marathi (the local language), and were both willing to participate in the study (*n* = 1201). Further details on the original study rationale, setting, methods, and intervention are available elsewhere [24].

The present study used data from a cross-sectional survey that was conducted after the final follow-up survey (18 months post-baseline) of CHARM2 was completed. The CHARM2 study was conducted between October 2018 and December 2020. At the 18-month follow-up assessment in June–December 2020, participants were asked whether investigators could maintain their contact information to invite them into future research projects. More than 90% of participants confirmed their willingness to be re-contacted for additional research opportunities. These couples formed the sample pool for the cross-sectional survey that was conducted between 1 February and 31 March 2021 after the state government-imposed lockdown was lifted in the state. The survey sought to investigate the impact of the COVID-19 pandemic on health outcomes and the role of gender inequities and socio-economic stressors. In this survey, at least one member of each of the 1028 couples provided complete surveys, representing an 85% retention rate relative to the initial CHARM2 recruited population. The analytic sample for this study was comprised of 1021 women and 1020 men who had non-missing information for all study variables.

### 2.2. Measures

#### 2.2.1. Mental Health Symptoms during the COVID-19 Pandemic

We adapted questions from the World Health Organization World Mental Health Composite International Diagnostic Interview (WHO WMH-CIDI) [25]. The CIDI enables interviewers to assess mental disorders as per the ICD-10 and DSM classifications. We included nine items from the screening section, which included a symptom checklist for different mental health issues including depression, anxiety, and panic (e.g., Have you ever in your life had a period of time lasting several days or longer when most of the day you felt sad, empty, or depressed?). The response options were: yes, both before and during the COVID-19 pandemic; yes, only during the COVID-19 pandemic; yes, only before the COVID-19 pandemic; and no, never. In this study, we wanted to isolate the effect of the pandemic on mental health symptoms from other prior causes of mental health symptoms. Therefore, we focused on respondents who reported mental health symptoms only during the COVID-19 pandemic and not before the pandemic. Consequently, each item was recoded into a binary variable (0 = no, never, 1 = only during the COVID-19 pandemic). In addition, each item was recoded to missing if respondents reported experiencing a symptom only before the COVID-19 pandemic or both before and during the COVID-19 pandemic to exclude observations from respondents who had reported experiencing the symptom before the pandemic. We summed these dichotomized items to obtain a measure of the number of mental health symptoms that were reported as having occurred only during the COVID-19 pandemic. Cronbach’s alpha was 0.85 for women and 0.78 for men in this study. The WHO WMH-CIDI was used in India in prior studies and was found to be reliable [26,27].

#### 2.2.2. Financial Hardship

A single item was used to measure financial hardship. Participants were asked: Have you had financial hardship (not enough money for food or other basic needs) due to the COVID-19 pandemic? The response options were yes/no.

#### 2.2.3. Job Loss

Participants who reported that they had been employed before the pandemic were asked if they were able to work as usual once the pandemic started. The response options were: yes; no, but I was provided my wages in full; no, but I was provided with partial wages; and no, and I received no pay. This item was recoded into a binary variable (0 = worked as usual or work changed but received full or partial wages, 1 = work changed and received no wages).

#### 2.2.4. Social Support

We used eight items from the Medical Outcomes Study (MOS) Social Support Survey [28] to assess participants’ experiences of social support. Participants were asked how often someone was available to help them with eight tasks, such as taking them to the doctor, preparing meals, and socializing. The response options ranged from 1 = none of the time to 4 = most of the time. Higher scores indicated higher social support. Cronbach’s alpha was 0.89 for women and 0.94 for men in this study. The MOS was used in prior studies in India [29] and Thailand [30].

#### 2.2.5. Government Monetary Support

Participants were asked if they had received government support in the form of direct monetary payment to help their family deal with financial hardships due to the COVID-19 pandemic (0 = no, 1 = yes). Under the government scheme “Pradhan Mantri Kisan Samman Nidhi Yojana,” the central government provides small and marginal farmers with combined landholding of up to 2 hectares with annual income support of INR 6000. During the COVID-19 pandemic, farmers were paid their second installment earlier in the year to mitigate the effects of the pandemic.

#### 2.2.6. Government Resource Support

Participants were asked whether they had received government support in the form of food or other resources to help their family deal with financial hardships due to the COVID-19 pandemic (0 = no, 1 = yes). The National Food Security Act provides families whose household incomes fall below the government-specified poverty threshold with food grains at Rs 2 per kg for wheat and Rs 3 per kg for rice. Each family is allotted 35 kg of subsidized food grains per month under this scheme. Landless agricultural laborers, marginal farmers, artisans, and daily wage workers are also among the economically vulnerable eligible for subsidized food grains. During the COVID-19 pandemic, the monthly allocation was doubled and families were provided 5 kg of wheat/rice and 1 kg of pulses free of cost under the government scheme “Pradhan Mantri Garib Kalyan Yojana.”

#### 2.2.7. Household SARS-CoV-2 Infection

Participants were asked whether any individual in their household was tested for SARS-CoV-2 infection and diagnosed positive (0 = no, 1 = yes).

#### 2.2.8. SC/ST/OBC

Participants were asked to denote their caste, where the caste status variable was recoded as a binary variable to identify participants who belonged to a scheduled caste (SC), scheduled tribe (ST), or other backward class (OBC) (0 = no, 1 = yes). SC/ST/OBC individuals are socioeconomically minoritized groups in India.

#### 2.2.9. Poverty

Below Poverty Line (BPL) cards are issued to families whose household incomes fall under the state-specified poverty threshold. Households use these BPL cards to access subsidized grains, cereals, and sugar under the National Food Security Act. Ownership of BPL cards was used as a proxy for poverty and measured as a binary variable (0 = no, 1 = yes).

### 2.3. Analysis

We first assessed the demographic characteristics of all participants and conducted bivariate regression analysis between these factors and mental health symptoms during the COVID-19 pandemic. These results are presented in Table 1. Demographic questions regarding age, education, and caste were collected at the baseline of the CHARM2 evaluation, which took place approximately 2 years prior to the survey under consideration. We added two years to the age variable to reflect the current age of our participants. We used data on the number of children, household income, and poverty status from the 18-month survey that was conducted 9 months before the current COVID-19 survey. We also assessed descriptive statistics for all items that measured mental health symptoms (Table 2). After completing the descriptive analysis, we used multilevel models to test the associations between financial hardship, job loss, and mental health symptoms. We stratified the sample by gender and estimated separate models for women and men. Models that included the independent variable of job loss were restricted to 350 women and 317 men who reported that they were employed before the start of the pandemic. Mental health symptoms were measured as a count variable and we used multilevel Poisson models to examine the associations between financial hardship, job loss, and mental health symptoms, including a random intercept for the subcenter, the unit of randomization. All models were adjusted for age, number of children, caste, poverty, education, social support, receipt of government support in the form of food or other resources, receipt of monetary support from the government, and whether any individual in the household tested SARS-CoV-2 positive. We created another variable to measure any prior experience of mental health but it was not significantly associated with mental health symptoms in the bivariate analysis (results not shown). Otherwise, we would have included it as a covariate for sensitivity analyses.

### 2.4. Ethical Approval

Ethical approval for this study was obtained from the University of California San Diego and the Sigma Institutional Review Board in India. Written consent was obtained from all participants; illiterate participants could have the form read to them by the study personnel or a trusted person of their choosing.

## 3. Results

### 3.1. Descriptive Analysis

#### 3.1.1. Sample

On average, women were 24 years old at the baseline (Table 1). Most women were educated: 29% had completed secondary education, 27% had completed higher secondary, and 32% completed post-secondary or higher education. Approximately one-third of women (29%) identified as SC/ST/OBC. More than half the sample (56%) had experienced financial hardship due to the pandemic. Thirty-four percent of women were working prior to the start of the pandemic. Among women who were employed at the beginning of the pandemic, 21% reported job loss due to the pandemic. Eleven percent of women reported that a family member had tested positive for SARS-CoV-2 infection. While most women did not report a need to access health services (68%), 29% needed care and were able to access it, while 3% were unable to access the health services they needed. A large proportion of the sample (88%) reported receiving government support in the form of resources, such as food during the pandemic. Most women reported moderate levels of social support (M = 18.85, range = 0–32). On average, women reported experiencing four (of nine) mental health symptoms only during the COVID-19 pandemic.

On average, men were 30 years old at baseline (Table 1). A majority of men were educated, with 31% having completed secondary education, 26% having completed higher secondary and 30% having completed post-secondary or higher education. On average, couples had one child. Thirty percent of male respondents belonged to SC/ST/OBC communities and 24% reported that they were living in poverty, as indicated by Below Poverty Line (BPL) card ownership. Sixty-one percent of men reported experiencing financial hardship due to the pandemic. Among the men who were employed at the start of the pandemic, 52% reported job loss due to the pandemic. Eleven percent of men reported that a household member tested positive for SARS-CoV-2 infection. Most men did not need health services (66%); among those who needed health services, almost all (33%) were able to do so, while <1% were unable to access the health services they needed.

#### 3.1.2. Mental Health Symptoms during the COVID-19 Pandemic

Table 2 presents a percentwise breakdown of the responses for each of the nine mental health symptoms. Most women (73%) and men (66%) experienced a symptom of panic disorder (item 1), followed by symptoms of depression (items 2–4) and a symptom of generalized anxiety disorder (item 7) only during the COVID-19 pandemic. Sixty-five percent of women and 54% of men reported feeling sad, empty, or depressed only during the pandemic. Most of the sample reported an onset of mental health symptoms only during the COVID-19 pandemic. Less than one percent of men and approximately 1–4% of women had experienced mental health symptoms only before the pandemic. Similarly, approximately 1–3% of women and <1% of men reported symptoms both before and during the COVID-19 pandemic. Across all items, a higher percentage of men (34–94%) reported no experience of mental health symptoms as compared to women (23–82%).

### 3.2. Multivariate Analysis

Multilevel regression models that assessed the adjusted associations between financial hardship and mental health symptoms showed that women who reported experiencing financial hardship had 1.29 times the rate of reporting mental health symptoms as women who were not experiencing financial hardship (aIRR = 1.29, C.I.: 1.21–1.39) (Table 3). Women with a household member who tested positive for SARS-CoV-2 infection had a higher rate of reporting mental health symptoms (aIRR = 1.35, C.I.: 1.23–1.48). SC/ST/OBC women reported higher rates of mental health symptoms than women who were not SC/ST/OBC (aIRR = 1.08, C.I.: 1.01–1.16). Women who were experiencing poverty had 1.10 times the rate of reporting mental health symptoms as compared to women who were not experiencing poverty (aIRR = 1.10, C.I.: 1.02–1.18). Women who needed to access health services and were able to do so were more likely to report mental health symptoms as compared to women who did not need to access health services (aIRR = 1.18, C.I.: 1.10–1.26). Social support was associated with a lower rate of reporting of mental health symptoms (aIRR = 0.98, C.I.: 0.97–0.99).

Among men, there was no association between financial hardship and reporting mental health symptoms (Table 3). Men who lived in households where at least one person tested positive for SARS-CoV-2 infection had 1.36 times the rate of reporting mental health symptoms as compared to men with no household members who contracted a SARS-CoV-2 infection (aIRR = 1.36, C.I.: 1.21–1.52). Men who identified as SC/ST/OBC had 1.12 times the rate of reporting mental health symptoms as compared to men who did not identify as SC/ST/OBC (aIRR = 1.12, C.I.: 1.03–1.22). Men who needed to access health services and were able to do so had a higher rate of reporting mental health symptoms than men who did not need to access health services (aIRR = 1.33, C.I.: 1.21–1.45). The number of children was negatively associated with mental health symptoms (aIRR = 0.93, C.I.: 0.88–0.99). Men who had post-secondary or higher education had a lower rate of reporting mental health symptoms as compared to men who had primary or no education (aIRR = 0.85, C.I.: 0.75–0.97).

We tested the association between job loss and mental health symptoms among women and men who were employed before the pandemic; the results are presented in Table 4. Among women, the association between job loss and mental health symptoms was not statistically significant. Similar to the other model, women who reported that a household member tested positive for SARS-CoV-2 infection (aIRR = 1.58, C.I.: 1.33–1.87) and those who needed to access health services and were able to do so were more likely to report mental health symptoms (aIRR = 1.31, C.I.: 1.14–1.50). Women who received government support in the form of resources, such as food, had lower rates of reporting mental health symptoms (aIRR = 0.76, C.I.: 0.61–0.93).

Among men who were working before the start of the pandemic, men who lost their jobs or wages had 1.16 times the rate of reporting mental health symptoms as compared to men who did not report job loss (aIRR = 1.16, C.I.: 1.10–1.34) (Table 4). Men who needed to access health services and were able to do so had a higher rate of reporting mental health symptoms than men who did not need to access health services (aIRR = 1.42, C.I.: 1.22–1.67).

## 4. Discussion

This study examined the association between COVID-19-related financial hardship, job loss and mental health symptoms during the COVID-19 pandemic in a rural area in Maharashtra approximately six months after the first lockdown took place in India. Women reported more novel onset mental health symptoms during the COVID-19 pandemic than men (4.2 vs. 2.8). The majority of study respondents reported financial hardship subsequent to the first COVID-19 lockdown in India (56% of women and 60.7% of men). Job loss was reported by more than one-fifth of women (21.4%) and more than half of men (51.7%). We found a gender difference in the association between the two independent variables in our study, financial hardship and job loss, and the dependent variable of mental health symptoms. Financial hardship was associated with poor mental health symptoms among women whereas job loss was associated with poor mental health symptoms among men. These findings align with prior research conducted outside of India that suggested that job loss and financial hardship were associated with poor mental health during the COVID-19 pandemic [6] and recessions [19,20].

Our results also extend the understanding of these relationships by suggesting that financial hardship may tend to affect women’s mental health, while job loss may impact men’s mental health, aligning with findings from a systematic review that found that men were more likely to experience poor mental health as a result of unemployment as compared to women [31]. Research suggests that men are more likely to feel isolated [32] and experience a higher loss of self-esteem [33] due to job loss as compared to women. Loss of employment can be stigmatizing for men in patriarchal societies, such as India, where norms of masculinity are intricately linked to employment status [34]. Male employment may also have a larger impact on a family’s economic situation, as men are paid higher wages, especially in rural areas, and most families in India rely on wages from men rather than women [35,36]. Additional qualitative research is warranted to better understand the mechanisms that underlie the gender differences in the association between financial stressors and mental health symptoms.

Our study shows that social support was associated with a lower likelihood of reporting mental health symptoms among women. This is in line with existing research suggesting that social support is positively associated with mental health, and can mitigate the negative impacts of unemployment [37]. Studies also found that women were more vulnerable to experiencing mental health issues without adequate social support when experiencing unemployment and other negative life events as compared to men [38,39]. It should be noted that the association between social support and mental health symptoms was not significant among women who were employed at the beginning of the pandemic. Rather, among employed women, government support in the form of free/subsidized food was positively associated with lower reporting of poor mental health symptoms, suggesting that the modalities of support that are most useful to women’s mental health may differ based on their employment and financial status. Nevertheless, it is important to promote both social support and social protection initiatives in tandem to address the mental health vulnerabilities of women from different financial security statuses.

Health concerns were also associated with poor mental health among women and men. For both groups, a household member’s positive SARS-CoV-2 infection diagnosis was associated with poor mental health. Women and men who accessed health services were also more likely to report poor mental health. The health system in India was severely overburdened during the COVID-19 pandemic and there was an acute shortage of hospital beds, ventilators, oxygen, and other services [2,3,40,41]. It is probable that several factors, including lower-quality care, unavailability of certain services, high cost of health services during the pandemic, disruption in wage work, or concern for their own or their family member’s health affected respondents’ mental health.

While the economic fallout of the pandemic has been severe, they have not been shared equitably across caste groups. In our sample, SC/ST/OBC women and men were more likely to report mental health symptoms. Lower educated individuals were also more likely to experience mental health issues. Studies found that generous government assistance can buffer individuals from unemployment and poor mental health in the United Kingdom, United States, and Europe [22]. There is an urgent need for policy solutions that address structural flaws in the Indian economy that disproportionately impact minoritized groups. Policymakers should scale up the financial assistance that was provided during the lockdown under the Pradhan Mantri Garib Kalyan Yojana, which included free and/or subsidized grains, since receiving this aid was associated with better mental health in our study. In addition, the government can link food and cash assistance programs to cyclical changes in the economy to ensure that marginalized groups are not left out of the recovery process. There is also a need for different kinds of job creation programs that range from improving access to short-term employment, such as the Mahatma Gandhi National Rural Employment Guarantee Act, 2005 (MNERGA), which has aided individuals who lost jobs in the pandemic [16], to subsidies for employment sectors that were disproportionately affected by the pandemic to a central government jobs guarantee program that can provide long-term guaranteed employment.

Finally, we note that only two factors, social and resource support, were associated with lower levels of novel-onset mental health symptoms, neither of which was protective for men. Broader availability of mental health services, outreach, and norms shifting regarding the acceptability of discussing and using mental health services is needed to support individuals through, and beyond, this mental health crisis [42]. For example, at the study site, mental health services are not provided in the local public health center, which provides proximate and affordable care. Mental health professionals are located in big villages or cities and patients often have to travel between 2 and 30 km to access their services depending on their location. There is an urgent need to improve the availability and affordability of mental health services in rural areas. To do so, mental health needs to be integrated into primary health care by improving the mental health literacy of primary health care workers who are often the first point of contact for rural communities [43]. Mental health professionals could also set up a consultation service for rural primary health care workers who care for patients presenting with mental health symptoms [44].

### Limitations

The present study used cross-sectional data and, therefore, no causal inferences could be made. We only asked respondents who were engaged in work for pay prior to the start of the pandemic whether they had lost their job or wages during the pandemic. In our sample, approximately one-third of men (31%) and women (34%) reported that they worked for pay before the pandemic began. Additionally, we did not ask participants about the nature of their job prior to the start of the pandemic. Because our sample was from a rural agrarian community, the low rates of employment before the start of the pandemic may reflect seasonal variations in agricultural labor. Future studies can examine differences in the association between job loss and mental health symptoms by season and sector of employment. We measured education in the first wave of the original CHARM2 study in 2018 and the participants’ education status may have changed in the interim. We used two measures that were used in prior studies in India but have not been validated. Further, we used a composite measure of mental health symptoms, which did not allow us to compare the differential impact of financial hardship and job loss on different kinds of mental health disorders. Future studies can use validated measures of mental health symptoms for a range of disorders and social support. However, it is important to note that we did a pilot test of our measures to ensure cultural appropriateness and comprehension. All data are self-reported and are therefore subject to recall and social desirability biases. Our results are limited to a rural population in a single state and, therefore, our study results cannot be generalized to the larger population in India. The findings suggest that a nationwide study to assess the regional differences in the relationship between financial stressors and mental health in India may be warranted.

## 5. Conclusions

Our study presents evidence that highlights the association between financial stressors and mental health during a global pandemic. Financial hardship was associated with poor mental health symptoms among women and job loss was associated with poor mental health symptoms among men. There is a need for policy and programmatic initiatives that can bolster early detection of and intervention with individuals who are at an increased risk of experiencing mental health issues. Future studies can focus on developing and testing interventions that can create awareness of mental health issues and provide mental health support services to populations that have been adversely impacted by the financial fallout of the COVID-19 pandemic. There is also an urgent need for financial assistance and job creation programs, especially for minoritized populations, to aid families in the recovery process.

## Figures and Tables

**Table 1:** Descriptive statistics of participants.

Variable	Women (*n* = 1021)			Men (*n* = 1020)		
	*n* or Mean	% or SD	p *	*n* or Mean	% or SD	p *
Mental health symptoms	4.05	2.84		2.82	2.3	
Financial hardship	572	56.02%	**0.00**	620	60.78%	0.53
Work for pay prior to COVID-19 pandemic	350	34.28%		317	31.08%	
Job loss	75	21.43%	**0.00**	164	51.74%	**0.00**
Age	23.96	2.97	0.72	29.60	3.69	0.74
Attended school	1009	98.82%	**0.03**	1017	99.71%	**0.02**
Education						
No education/primary	126	12.40%	**0.01**	134	13.20%	0.07
Secondary	296	28.99%		310	30.50%	
Higher secondary	275	26.93%		270	26.49%	
Post-secondary or higher	324	31.73%		306	29.81%	
Number of children	1.45	0.71	**0.02**	1.45	0.71	**0.03**
Caste						
None/other	718	70.31%	**0.00**	710	69.61%	**0.01**
SC/ST	303	29.69%		310	30.39%	
Income (INR)	18,003.38	20,834.67	0.95	26,054	55,037.59	0.48
Poverty	245	24.00%	**0.00**	245	24.02%	0.20
Household member tested SARS-CoV-2 positive	109	10.64%	**0.00**	109	10.69%	**0.00**
Treatment assignment for original trial						
Control	515	50.59%	0.70	515	50.49%	0.37
Intervention	506	49.41%		505	49.51%	
Access to health services						
No need	691	67.77%	**0.00**	676	66.27%	**0.00**
Yes, had access	302	29.49%		341	33.33%	
Yes, unable to access	28	2.73%		3	0.29%	
Government monetary support	114	11.13%	0.50	114	11.14%	0.07
Government resource support	900	88.18%	**0.01**	899	88.14%	0.22
Social support	18.85	4.82	**0.00**	16.31	5.99	0.55

* Results from bivariate regressions with respondent’s reported mental health symptoms. Bold *p*-values represent statistical significance.

**Table 2:** Percentwise distribution of items that measured mental health symptoms.

No.	Item	Never		Only during the COVID-19 Pandemic		Only before the COVID-19 Pandemic		Both before and during the COVID-19 Pandemic	
		% Women	% Men	% Women	% Men	% Women	% Men	% Women	% Men
1	Have you ever in your life had an attack of fear or panic when all of a sudden you felt very frightened, anxious, or uneasy?	23.21	33.82	72.67	65.78	2.06	0.29	2.06	0.1
2	Have you ever in your life had a period of time lasting several days or longer when most of the day you felt sad, empty, or depressed?	31.44	45.29	64.74	54.12	1.47	0.39	2.35	0.2
3	Have you ever had a period of time lasting several days or longer when most of the day you were very discouraged about how things were going in your life?	49.36	56.37	47.6	43.24	1.96	0.2	1.08	0.2
4	Have you ever had a period of time lasting several days or longer when you lost interest in most things you usually enjoy?	60.82	68.43	38	31.27	0.78	0.29	0.39	0
5	Have you ever had a period of time lasting four days or longer when most of the time you were very irritable, grumpy, or in a bad mood?	51.91	80.29	41.63	19.51	3.62	0.1	2.84	0.1
6	Have you ever had a period of time lasting four days or longer when most of the time you were so irritable that you either started arguments, shouted at people, or hit people?	82.17	93.53	16.45	5.78	0.69	0.69	0.69	0
7	Did you ever have a time in your life when you were a “worrier,” that is, when you worried a lot more about things than other people with the same problems as you?	55.14	69.51	43.1	29.9	0.88	0.49	0.88	0.1
8	Did you ever have a time in your life when you were much more nervous or anxious than most other people with the same problems as you?	67.29	78.73	30.36	20.88	1.57	0.2	0.78	0.2
9	Did you ever have a period lasting one month or longer when you were anxious and worried most days?	47.11	87.55	50.44	12.35	0.78	0.1	1.67	0

**Table 3:** Multivariate associations between financial hardship and mental health symptoms by gender.

Variable	Model 1	Model 2
	Women (*n* = 1021)	Women (*n* = 1020)
	aIRR	aIRR
Financial hardship	1.29 ***	0.99
	(1.21–1.39)	(0.91–1.09)
Household SARS-CoV-2 infection status	1.35 ***	1.36 ***
	(1.23–1.48)	(1.21–1.52)
Age	1.00	1.00
	(0.99–1.01)	(0.99–1.01)
SC/ST/OBC	1.08 *	1.12 *
	(1.01–1.16)	(1.03–1.22)
Number of children	1.04	0.92 **
	(0.99–1.10)	(0.87–0.98)
Education (reference category: primary or no education)		
Secondary	0.98	0.98
	(0.89–1.09)	(0.86–1.10)
Higher secondary	0.94	0.88
	(0.85–1.05)	(0.77–1.00)
Post-secondary or higher	0.95	0.85 *
	(0.85–1.06)	(0.75–0.97)
Poverty status	1.10 *	1.03
	(1.02–1.18)	(0.94–1.13)
Treatment group	1.26	1.21
	(0.71–2.24)	(0.78–1.89)
Access to health services (reference category: no services needed)		
Accessed health services	1.18 ***	1.33 ***
	(1.10–1.26)	(1.21–1.45)
Unable to access health services	1.21	1.72
	(1.00–1.46)	(0.99–3.00)
Government monetary support	0.98	1.13
	(0.88–1.08)	(0.98–1.30)
Government resource support	0.92	0.96
	(0.84–1.01)	(0.84–1.11)
Social support	0.98 ***	1.00
	(0.97–0.99)	(0.99–1.02)
Number of groups	20	20

Note: *** *p* < 0.001, ** *p* < 0.01, * *p* < 0.05. Confidence intervals are given in parentheses. All models were adjusted for age, number of children, caste, poverty, education, social support, receipt of government support in the form of food or other resources, receipt of monetary support from the government, and whether any individual in the household tested SARS-CoV-2 positive.

**Table 4:** Multivariate associations between job loss and mental health symptoms by gender.

Variable	Model 1	Model 2
	Women (*n* = 350)	Men (*n* = 317)
	aIRR	aIRR
Job/wage loss	1.19	1.16 *
	(0.94–1.50)	(1.01–1.34)
Financial hardship	1.30 ***	1.07
	(1.15–1.47)	(0.92–1.25)
Household SARS-CoV-2 infection status	1.58 ***	1.17
	(1.33–1.87)	(0.95–1.44)
Age	1.00	1.01
	(0.98–1.02)	(0.99–1.03)
SC/ST/OBC	1.04	0.98
	(0.90–1.20)	(0.85–1.14)
Number of children	1.12 **	0.95
	(1.03–1.21)	(0.86–1.05)
Education (reference category: primary or no education)		
Secondary	0.95	1.01
	(0.81–1.12)	(0.83–1.24)
Higher secondary	0.93	0.86
	(0.78–1.12)	(0.69–1.08)
Post-secondary or higher	0.85	0.89
	(0.70–1.02)	(0.72–1.11)
Poverty status	1.11	1.00
	(0.98–1.24)	(0.86–1.17)
Treatment group	1.21	1.08
	(0.73–2.00)	(0.74–1.56)
Access to health services (reference category: no services needed)		
Accessed health services	1.31 ***	1.42 ***
	(1.14–1.50)	(1.22–1.67)
Unable to access health services	1.09	1.63
	(0.76–1.56)	(0.62–4.25)
Government monetary support	0.88	1.05
	(0.76–1.03)	(0.79–1.40)
Government resource support	0.76 **	0.80
	(0.61–0.93)	(0.61–1.05)
Social support	0.99	1.00
	(0.98–1.00)	(0.98–1.02)
Number of groups	20	20

Note: *** *p* < 0.001, ** *p* < 0.01, * *p* < 0.05. Confidence intervals are given in parentheses. All models were adjusted for age, number of children, caste, poverty, education, social support, receipt of government support in the form of food or other resources, receipt of monetary support from the government, and whether any individual in the household tested SARS-CoV-2 positive.

## Data Availability

The data used for this study are not publicly available to maintain the privacy of research participants.
